# Survival and risk factors associated with surgical repair of ventricular septal rupture after acute myocardial infarction: A single-center experience

**DOI:** 10.3389/fcvm.2022.933103

**Published:** 2022-07-19

**Authors:** Keyan Zhao, Baoyin Li, Biao Sun, Dengshun Tao, Hui Jiang, Huishan Wang

**Affiliations:** Department of Cardiovascular Surgery, General Hospital of Northern Theater Command, Shenyang, China

**Keywords:** acute myocardial infarction, ventricular septal rupture, surgery, risk factor, survival

## Abstract

**Objective:**

To analyze the survival and risk factors associated with the surgical treatment of ventricular septal rupture (VSR) after acute myocardial infarction (AMI).

**Methods:**

We retrospectively analyzed 45 consecutive patients with VSR after AMI whose procedures were performed in the Department of Cardiovascular Surgery at the General Hospital of Northern Theater Command between January 2012 and December 2021. Relevant clinical data, surgery-related conditions, and follow-up data of all patients were summarized. Patients were divided into the survival group and the death group. The Kaplan–Meier method and log-rank test were used to determine the cumulative incidence of all-cause mortality. Multivariate logistic regression was used to evaluate the independent risk factors for all-cause mortality.

**Results:**

The average postoperative follow-up time was 42.1 ± 34.1 months. The overall mortality rate was 20% (9/45 patients) and the operative mortality rate was 8.9% (4/45 patients). Logistic analysis showed that the death group had higher serum creatinine (127.32 ± 47.82 vs. 82.61 ± 27.80 μmol/L, respectively; *P* = 0.0238) and N-terminal pro-B-type natriuretic peptide (NT-proBNP) [8,654.00 pg/mL (6,197.00–11,949.00 pg/mL) vs. 4,268.96 pg/mL (1,800.00–7,894.00 pg/mL), respectively; *P* = 0.0134] levels than the survival group. The cardiopulmonary bypass time (CPB) was longer in the death group than in the survival group [131.00 min (121.00–184.00 min) vs. 119.00 min (103.00–151.50 min), respectively; *P* = 0.0454]. Significantly more red blood cells were transfused in the death group than in the survival group [11.60 units (6.10–16.50) vs. 3.75 units (0.00–7.00 units), respectively; *P* = 0.0025]. Intra-aortic balloon pump (IABP) implantation (*P* = 0.016) and ventilation time (*P* = 0.0022) were risk factors for mortality. A 1-month landmark analysis showed that compared with patients with VSR to surgical time >14 days, patients who underwent surgery within 14 days had a higher rate of all-cause mortality (25.00 vs. 3.33%; log-rank *P* = 0.023). Patients with VSR within 14 days also had a higher rate of residual shunts that were higher than moderate. Multivariate analysis showed that transfusion of red blood cells and NT-proBNP level were risk factors for all-cause mortality, as well as major adverse cardiovascular and cerebrovascular events.

**Conclusions:**

Surgical repair resulted in good outcomes for patients with VSR after AMI. Patients with VSR to surgical time >14 days had a lower rate of all-cause mortality. Treatment strategies for VSR should be based on the patient's condition and comprehensively determined through real-time evaluation and monitoring.

## Introduction

Ventricular septal rupture (VSR) is a rare but potentially fatal complication of acute myocardial infarction (AMI). With the emergence of thrombolysis and primary percutaneous coronary intervention (PCI), the incidence of VSR has decreased to between 0.25 and 0.31% (1).

In 1957, the first surgically repaired case of VSR was reported by Cooley and colleagues (2). Although the mortality rate associated with AMI has improved over the course of the past two decades, the results of surgical repair for VSR are often suboptimal and associated with high mortality rates attributable to hemodynamic instability and tissue fragility (3). A retrospective review of the Society of Thoracic Surgeons national database between 1999 and 2010 found that the operative mortality rates were 54.1% if the repair was performed ≤ 7 days after myocardial infarction (MI) and 18.4% if the repair was performed >7 days after MI (4). Moreover, the mortality rate increases if cardiogenic shock develops before surgery. The precise timing of surgery depends on the patient's hemodynamic status (5).

This study systematically reviewed the surgical experience and follow-up results of 45 cases in our department during the previous 10 years. We performed a risk factor analysis to determine the best treatment strategy to improve the poor prognosis associated with VSR.

## Materials and methods

### General data

We retrospectively analyzed 45 consecutive patients who experienced VSR after AMI and underwent surgical treatment in our department between January 2012 and December 2021. AMI was diagnosed when typical chest pain, increased serum troponin T levels >14 ng/L, and electrocardiogram (EKG) evidence of >2 mm ST-segment elevation in precordial leads or >1 mm ST-elevation in limb leads were observed. VSR was diagnosed using echocardiography. All patients were evaluated using coronary angiography (CAG). When patients were admitted to our department, they were initially treated medically. We tried to manage severe heart failure and shock with an intra-aortic balloon pump (IABP) and extracorporeal membrane oxygenation (ECMO). If the patient's condition was unstable or worsened because of organ dysfunction, as indicated by a ventilator, dialysis, or increased liver enzymes, emergency surgery was considered. Patients were divided into the survival group and the death group. The primary outcome assessed was all-cause mortality, including operative death and follow-up death. Operative death was defined as any death, regardless of cause, occurring within 30 days after surgery (in or out of the hospital) or 30 days after the same hospitalization subsequent to surgery. Secondary endpoints were major adverse cardiovascular and cerebrovascular events (MACCE). This study conformed with the principles of the Declaration of Helsinki and was approved by the Ethics Committee of the General Hospital of Northern Theater Command.

### Surgery

All surgical procedures were performed under general anesthesia. Median sternotomy was performed for all patients. The left internal mammary artery or great saphenous vein was selected as the graft vessel for coronary artery bypass graft (CABG) according to the status of coronary artery disease. Cardiopulmonary bypass (CPB) with moderate hypothermia was commenced with ascending aortic perfusion and bicaval cannulations. The left ventricle was vented through the right superior pulmonary vein. Measures to ensure myocardial protection included cold blood antegrade and retrograde cardioplegia infusion. The left ventricle was opened approximately 1 to 2 cm parallel to the left anterior artery (LAD) or posterior descending artery (PDA) at the infarction zone or aneurysm. Ventricular septal dissection was diagnosed preoperatively by echocardiography, with key features including neocavitation within the myocardium, communication between the ventricles through the neocavitation, and detection of flow within the cavity. Surgical exploration could further confirm the diagnosis. If there was ventricular septal dissection, then the ventricular incision could be performed on the right of the LAD or PDA. A polyester patch (Shanghai Chest Medical Technology Co., Shanghai, China) was used to close the rupture, as described by Daggett et al. (6). Mattress sutures with interrupted pledgeted 4–0 prolene were stitched with a sufficient 1-cm margin around the VSR. The left ventricle was closed using the sandwich method regularly. The Dor procedure (7) was performed if an obvious aneurysm existed. When posterior VSR was diagnosed by echocardiography, the defect was explored first from the right atrial incision and the tricuspid valve orifice. If the VSR was located at the sinus interventricular septum with firm margins, it was closed from the right atrial incision. If this was not the case, we closed it using the left ventricular incision. Repair or replacement was performed if the mitral valve was incompetent.

Follow-up data were obtained by consulting the hospital medical records and telephoning patients or their families. Clinical examination and echocardiography results were recorded.

### Statistical methods

All data were statistically analyzed using SPSS 25.0 (IBM, Armonk, NY, USA). Normally distributed data are expressed as mean ± standard deviation, and non-normally distributed data are expressed as median (interquartile range). Count data are expressed as the frequency and percentage. Comparisons between baseline characteristics were performed using the Student *t*-test for distributed continuous variables, the Mann–Whitney test for skewed distributed continuous variables, and the chi-square test with Fisher's exact test for categorical variables. Multivariate logistic regression analyses were performed to determine the independent risk factors for all-cause mortality. First, we performed univariate analysis. Subsequently, significant factors identified by the univariate analysis and variables known a priori to be associated with the clinical outcome, such as time from VSR to surgery, were put in multivariate analysis. The regression variables included demographic characteristics, surgical information, and laboratory examination results. The Kaplan–Meier method was used to estimate the cumulative incidence of patient mortality. *P* <0.05 was considered statistically significant.

## Results

The general data and results are presented in Table 1. The average age of the patients was 63.58 ± 8.21 years, including 25 male patients (55.56%). The time from the onset of AMI to the diagnosis of VSR was 3.00 days (2.00–6.00 days), and the time from the diagnosis of VSR to surgery was 21.00 days (14.00–28.00 days). The diameter of the rupture was 1.79 ± 0.57 cm. The rupture was located at the anterior ventricular septum in 38 cases and at the posterior ventricular septum in seven cases. All patients underwent VSR repair with CPB under general anesthesia. Thirty-nine CABG procedures, 13 Dor procedures, 3 mitral replacements, and 2 tricuspid repairs were performed. The mean New York Heart Association grade was 3.38 ± 0.86. IABP was performed before surgery or at the time of surgery in 27 cases. ECMO was used because of shock before surgery in one case and during surgery in one case. The left ventricle ejection fraction was 0.46 ± 0.07, and the pulmonary systolic pressure was 61.24 ± 12.82 mmHg.

**Table 1:** Patient characteristics.

**Characteristics**	**All patients (*****N** =* **45)**	**Survival (*****N** =* **36)**	**Death (*****N** =* **9)**	***P*** **-value**
Age (years)	63.58 ± 8.21	63.36 ± 8.18	64.44 ± 8.79	0.7278
Male (%)	25 (55.56%)	19 (52.78%)	6 (66.67%)	0.7095
Weight (kg)	65.72 ± 9.37	65.48 ± 9.15	66.67 ± 10.75	0.7385
Diabetes mellitus, *n* (%)	14 (31.11%)	10 (27.78%)	4 (44.44%)	0.4275
Previous infarction, *n* (%)	1 (2.22%)	1 (2.78%)	0 (0.00%)	1
History of PCI, *n* (%)	13 (28.89%)	10 (27.78%)	3 (33.33%)	0.7037
History of smoking, *n* (%)	18 (40.00%)	1 3 (36.11%)	5 (55.56%)	0.4487
**NYHA heart function grade, *n* (%)**				0.1863
1	1 (2.22%)	0 (0.00%)	1 (11.11%)	
2	8 (17.78%)	7 (19.44%)	1 (11.11%)	
3	9 (20.00%)	8 (22.22%)	1 (11.11%)	
4	27 (60.00%)	21 (58.33%)	6 (66.67%)	
**Preoperative angiography**				0.2404
Single vessel disease, *n* (%)	22 (48.89%)	19 (52.78%)	3 (33.33%)	
Double vessel disease, *n* (%)	16 (35.56%)	13 (36.11%)	3 (33.33%)	
Triple vessel disease, *n* (%)	7 (15.56%)	4 (11.11%)	3 (33.33%)	
**Preoperative echocardiography**				
LVEF	0.46 ± 0.07	0.46 ± 0.07	0.46 ± 0.10	0.9378
LVESD (mm)	37.63 ± 4.80	37.55 ± 5.00	38.00 ± 4.04	0.8234
LVEDD (mm)	48.65 ± 4.51	48.53 ± 3.77	49.11 ± 6.92	0.8134
Left atria diameter (mm)	38.98 ± 5.17	39.35 ± 5.18	37.56 ± 5.17	0.36
Serum creatinine (μmol/L)	91.55 ± 36.84	82.61 ± 27.80	127.32 ± 47.82	0.0238
NT-proBNP (pg/mL)	5,153.00 (2,165.74–9,587.50)	4,268.96 (1,800.00–7,894.00)	8,654.00 (6,197.00–11,949.00)	0.0134
Hemoglobin level (g/L)	116.07 ± 12.17	117.56 ± 10.55	110.11 ± 16.65	0.101
Time from AMI to VSR (days)	3.00 (2.00–6.00)	3.00 (2.00–4.00)	6.00 (3.00–7.00)	0.1867
Time from VSR to surgery (days)	21.00(14.00–28.00)	21.00(16.00–28.00)	23.00(13.00–27.00)	0.6085
Time from VSR to surgery				0.2188
≤ 14 days, *n* (%)	12(26.67%)	8(22.22%)	4(44.44%)	
>14 days, *n* (%)	33(73.33%)	28(77.78%)	5(55.56%)	
Pulmonary systolic pressure (mmHg)	61.24 ± 12.82	62.25 ± 13.05	57.22 ± 11.66	0.2978
CPB time (min)	121.00 (107.00–156.00)	119.00 (103.00–151.50)	131.00 (121.00–184.00)	0.0454
Aortic cross time (min)	69.00 (55.00–89.00)	64.00 (54.50–89.00)	78.00 (69.00–90.00)	0.2276
IABP implantation, *n* (%)	27 (64.29%)	18 (54.55%)	9 (100.00%)	0.016
ECMO, *n* (%)	2 (100.00%)	0 (0.00%)	2 (100.00%)	-
Ventilation time (h)	44.50 (39.50–92.00)	44.00 (25.50–70.00)	134.50 (62.00–216.00)	0.0022
Residual shunt, *n* (%)	7 (15.91%)	4 (11.11%)	3 (33.33%)	0.1307
**Shunt volume**				0.038
None, *n* (%)	38 (84.44%)	32 (88.89%)	6 (66.67%)	
Mild, *n* (%)	5 (11.11%)	4 (11.11%)	1 (11.11%)	
Moderate, *n* (%)	1 (2.22%)	0 (0.00%)	1 (11.11%)	
Severe, *n* (%)	1 (2.22%)	0 (0.00%)	1 (11.11%)	
**Location of VSR**				1
Anterior, *n* (%)	38 (84.44%)	30 (83.33%)	8 (88.89%)	
Posterior, *n* (%)	7 (15.56%)	6 (16.67%)	1 (11.11%)	
Diameter (cm)	1.79 ± 0.57	1.74 ± 0.52	2.01 ± 0.70	0.195
Transfusion of RBC (units)	4.00 (1.50–8.50)	3.75 (0.00–7.00)	11.60 (6.10–16.50)	0.0025
Use of LIMA, *n* (%)	10 (22.22%)	9 (25.00%)	1 (11.11%)	0.659
**Grafts**				0.4038
0, *n* (%)	4 (8.89%)	3 (8.33%)	1 (11.11%)	
1, *n* (%)	27 (60.00%)	23 (63.89%)	4 (44.44%)	
2, *n* (%)	10 (22.22%)	6 (16.67%)	4 (44.44%)	
3, *n* (%)	3 (6.67%)	3 (8.33%)	0 (0.00%)	
4, *n* (%)	1 (2.22%)	1 (2.78%)	0 (0.00%)	

*Data are presented as mean ± standard deviation, mean (range), or number (%).*

*AMI, acute myocardial infarction; IABP, intra-aortic balloon pump; CPB, cardiopulmonary bypass; ECMO, extracorporeal membrane oxygenation; LIMA, left internal mammary artery; LVEDD, left ventricular end-diastolic dimension; LVEF, left ventricular ejection fraction; LVESD, left ventricular end-systolic dimension; NT-proBNP, N-terminal pro-B-type natriuretic peptide; NYHA, New York Heart Association; PCI, percutaneous coronary intervention; RBC, red blood cell; VSR, ventricular septal rupture. P values in bold are considered significantly different (P <0.05)*.

**Figure 1:**
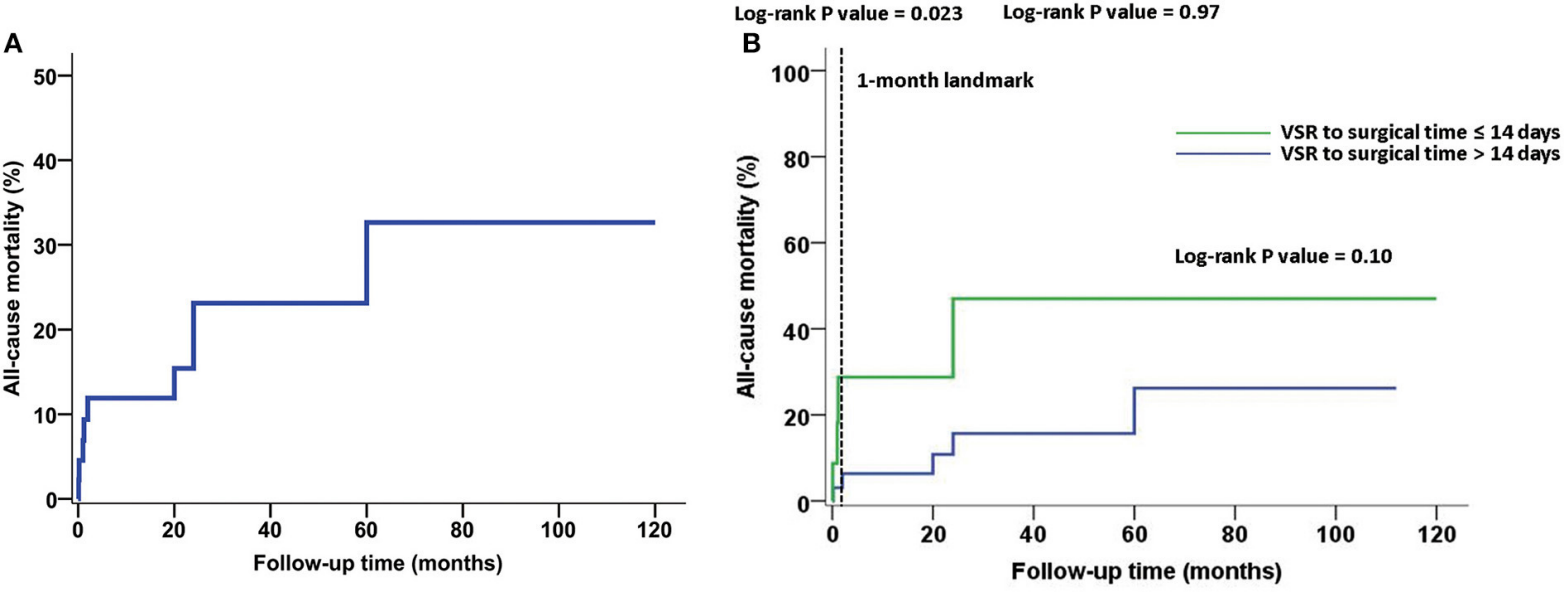
All-cause mortality during follow-up. **(A)** The Kaplan–Meier survival curve. **(B)** The time between ventricular septal rupture (VSR) and repair was not associated with all-cause mortality at ≤ 14 days and >14 days (33.33 vs. 15.15%; log-rank *P* = 0.10). However, the 1-month landmark analysis showed that compared to patients with VSR to surgical time > 14 days, those who underwent surgery within 14 days had a higher rate of all-cause mortality (25.00 vs. 3.33%; log-rank *P* = 0.023).

The average postoperative follow-up time was 42.1 ± 34.1 months. The overall and operative mortality rates were 20% (9/45 patients) and 8.9% (4/45 patients), respectively. The Kaplan–Meier survival curve is shown in Figure 1A. Logistic analysis showed that the serum creatinine level was higher in the death group than in the survival group (127.32 ± 47.82 vs. 82.61 ± 27.80 μmol/L, respectively; *P* = 0.0238). The N-terminal pro-B-type natriuretic peptide (NT-proBNP) level was also higher in the death group than in the survival group [8,654.00 pg/mL (6,197.00–11,949.00 pg/mL) vs. 4,268.96 pg/mL (1,800.00–7,894.00 pg/mL), respectively; *P* = 0.0134]. The CPB time was longer in the death group than in the survival group [131.00 min (121.00–184.00 min) vs. 119.00 min (103.00–151.50 min), respectively; *P* = 0.0454]. Significantly more red blood cells (RBCs) were transfused in the death group than in the survival group [11.60 units (6.10–16.50 units) vs. 3.75 units (0.00–7.00 units), respectively; *P* = 0.0025]. Our results also reveal that IABP implantation (*P* = 0.016) and ventilation time (*P* = 0.0022) were risk factors for mortality. The mortality increased with the number of diseased coronary vessels (from 3/22 in vessel to 3/16 in vessels and 3/7 in vessels), but there was no statistical significance (*P* = 0.2404). The use of the left internal mammary artery was not associated with all-cause death (Table 1). At follow-up, cerebral hemorrhage occurred in two cases and cerebral infarction occurred in one case, all of which involved residual physical impairment. Two patients experienced non-fatal MI. The significant factors (serum creatinine level, NT-proBNP level, CPB time, transfusion of RBC, IABP implantation, and ventilation time) identified by the univariate analysis, and the time from VSR to surgery, were inserted in multivariate analysis. Multiple regression analysis showed that transfusion of RBC and NT-proBNP level were independent risk factors for all-cause mortality and MACCE (Table 2). The time between ventricular septal rupture (VSR) and repair was not associated with all-cause mortality at ≤ 14 days and >14 days (33.33 vs. 15.15%; log-rank *P* = 0.10). However, the 1-month landmark analysis showed that compared with patients who underwent surgery >14 days after VSR, those who underwent surgery within 14 days had a higher rate of all-cause mortality (25.00 vs. 3.33%; log-rank *P* = 0.023) (Figure 1B). Echocardiography was used to assess residual shunts for grading of lesions as follows: mild shunt based on systolic color Doppler jet (width ≤ 3 mm); moderate shunt based on systolic color Doppler jet (width 3–4 mm); and large shunt based on systolic color Doppler jet (width > 4 mm) (8). All shunts were located at the suture line of the proximal septum. Mild shunts were present in five cases, while shunts higher than moderate were present owing to dehiscence in two cases who underwent the procedure ≤ 14 days after VSR.

**Table 2:** Independent risk factors for mortality.

**Independent risk factor**	**OR (95%CI)**	***P*** **-value**
**All-cause death (*n =* 9)**		
Increase of NT-proBNP per 100 pg/mL	1.019 (1.001–1.037)	0.035
Transfusion of RBC per 1 unit	1.328 (1.096–1.610)	0.0038
**Operative death (*n =* 4)**		
Transfusion of RBC per 1 unit	1.189 (1.010–1.399)	0.0373
**Death at follow-up (*n =* 5)**		
Transfusion of RBC per 1 unit	1.238 (1.028–1.491)	0.0243
**MACCE (*n =* 14)**		
Increase of NT-proBNP per 100 pg/mL	1.017 (1.001–1.033)	0.0373
Transfusion of RBC per 1 unit	1.466 (1.144–1.879)	0.0025

*MACCE, major adverse cardiovascular and cerebrovascular events; NT-proBNP, N-terminal pro-B-type natriuretic peptide; RBC, red blood cells; OR, odds ratio; CI, confidence interval*.

## Discussion

Although VSR after AMI is uncommon, it is associated with high mortality rates. A retrospective review of 2,876 cases revealed an overall operative mortality rate of 42.9%. Patients who died within 30 days tended to be older, female, had higher serum creatinine level, and had markers of more severe clinical acuity (4). A recent large retrospective multicenter international cohort (CAUTION) study reported a similar result that the early mortality rate was 40.4%. The study was conducted from January 2001 to December 2019 at 26 different centers worldwide among 475 consecutive patients who underwent surgery for postinfarction VSR. Older age, preoperative cardiac arrest and percutaneous revascularization, and postoperative need for IABP or ECMO support were independently associated with early mortality (9). The operative mortality observed during our study was 8.9%, which is better than that reported in previous studies (4, 9–11). There are two likely explanations for this result. One is that the mean time to operation after VSR was 21 days, and the tissue around the rupture was firm enough to sew in most patients. Some patients were first admitted to other hospitals and arrived at our department late. The other explanation might be that most of the patients presented with anterior VSR, which has been described to be associated with better survival (10), leading to an overall lower mortality. The CPB time was longer in the death group than in the survival group [131.00 min (121.00–184.00 min) vs. 119.00 min (103.00–151.50 min), respectively; *P* = 0.0454]. The aortic cross time was also longer in the death group than in the survival group [78.00 min (69.00–90.00 min) vs. 64.00 min (54.50–89.00 min), respectively; *P* = 0.2276], but there was no statistical significance. In general, aortic cross time is influenced by operative complexity. There were 2 patients with mitral valve replacement (MVR) and 2 patients with tricuspid repairs in the survival group and 1 patient with MVR in the death group. Meanwhile, the number of grafts did not differ between the two groups (*P* = 0.4038). Therefore, the prolonged CPB time was owing to the need for longer assistance to wean the patients from CPB but not owing to concomitant procedures including CABG. The conclusion that the aortic cross time was not a risk factor might be limited by the relatively small number of patients and requires further study. Preoperative creatinine and NT-proBNP levels were risk factors for mortality of VSR repair in our study, similar to other cardiac surgery studies (12, 13). For patients with delayed operations, we could not wait till these indicators increased too much. Deterioration of organ dysfunction illustrated by these indicators suggested the need for surgery. The amount of RBC transfusion was independently associated with death and MACCE. However, the death group had comparable hemoglobin level to the survival group (110.11 ± 16.65 vs. 117.56 ± 10.55 g/L, respectively; *P* = 0.101). Therefore, we should focus on surgical hemostasis and protection of blood cells during operation, as well as on pathological coagulation disorder in the perioperative period to decrease transfusions.

The timing of surgery for patients with VSR is particularly important. As previous studies (11, 14) have shown, emergency/rescue surgical procedures are closely related to higher mortality rates for patients with VSR. Bleeding problems and residual shunts are not rare during the early stage of VSR. Delayed surgery yields better results compared with emergency surgery. According to the data from the Society of Thoracic Surgeons, the operative mortality rates were 54.1%, if patients underwent surgery within 7 days of AMI and 18.4% if the repair was performed >7 days after AMI (4). Surgery can be performed 3 to 4 weeks after medical optimization with inotropic and mechanical cardiac support if the patient is hemodynamically stable (5, 15). In our study, the VSR to surgical time ≤ 14 days was associated with a higher rate of all-cause mortality compared to >14 days and within 1 month (25.00 vs. 3.03%; log-rank *P* = 0.023). Additionally, VSR surgery within 14 days was associated with shunts higher than moderate, which were also risk factors for mortality. IABP can decrease the left-to-right shunt by reducing afterload, increasing the coronary flow, diminishing ventricular wall stress and oxygen demand (14), and producing fewer side effects. Therefore, our strategy was to perform elective surgery >14 days after VSR, if the patient could be maintained with support from IABP. When mechanical ventilation had to be applied after IABP placement, we preferred to perform surgery within a few days because of its potentially harmful effects, including pneumonia and atelectasis from prolonged immobility.

During the early stage after VSR, effective mechanical-assisted circulation not only improves the patient's circulatory condition but also helps to delay surgery and avoid emergency surgery. Of the mechanical auxiliary devices, IABP is a good choice for maintaining stable circulation (14). However, this method cannot always prevent circulatory collapse in some patients in critical conditions. Recent studies and reviews have suggested the adoption of more aggressive mechanical circulatory support devices, such as ECMO, in order to reach hemodynamic stabilization (even cardiogenic shock reversal) and possibly delayed surgery for unstable patients (16–19). We had limited experience with ECMO in two VSR cases. One case involved a 64-year-old woman who required emergency surgery because of circulatory collapse after IABP insertion, 1.5 days after VSR. In this case we could not stop CPB and had to apply ECMO. Four days later, ECMO was discontinued and IABP was used until postoperative day 28. Finally, the patient died of residual shunt and multi-organ failure. The other case involved a 58-year-old man with an implanted stent in the LAD because of acute coronary occlusion; however, VSR occurred 3 days later. Five days after VSR, shock occurred. A ventilator with IABP and dialysis was implemented for this patient; however, the circulation could not be improved. ECMO was used and emergency surgery was performed. Four days later, ECMO was discontinued. The patient was discharged but died of residual shunt and multi-organ failure 2 years later. Therefore, ECMO could be an additional consideration as an alternative to emergent surgery in case of worsening conditions of patients. However, more experiences should be accumulated in clinical practice.

We generally used Daggett's method (6) with a large polyester patch and mattress sutures with interrupted pledgeted 4–0 prolene for repair if the time of VSR was beyond 14 days; however, we used David's method (20) if the VSR time was within 14 days. In this condition, the infarcted myocardium was weak and eventually friable like a slough, thus it could not afford stitches. The ideal suture line should be sewed to the healthy myocardium, but sometimes it is difficult to recognize the boundary between the viable myocardium and infarcted area. In addition, the shape of the patch might not fit the left ventricle well in three-dimension. These could be the reasons that caused strong tension, risked tearing the suture line, and caused residual/recurrent shunt, which has been considered the most crucial risk factor for poor outcomes. The Dor procedure was used in 13 patients with large aneurysms, where the involvement of the free wall and of the septum was similar or nearly similar (21). While it was not necessary when there was a larger extent of septum involvement. The residual shunt was detected by echocardiography. Some studies have reported that approximately 50–60% of residual shunts were observed in postoperative VSR patients, but most did not need repeat surgery (14, 22). In our study, regarding VSR time beyond 14 days, only three of 33 cases had a mild shunt, but they did not need reoperation. However, of the 12 cases with VSR time within 14 days, there was one case each with a mild, moderate, and severe shunt (12 mm). Two patients refused to undergo reoperation and died of multi-organ failure 30 days and 2 years after surgery, respectively. In all cases, the shunt was located at the suture line of the proximal septum. This might have been caused by tissue fragility, patch tension, and difficult exposure. We used to apply an oversized patch and suture the proximal septum margin with Teflon felt pledgets to reduce shunts in operation. In patients with VSR and ventricular septal dissection, there were some advantages of incising the ventricular septal dissection to the right of the coronary artery. First, we could see the opening to both ventricles and choose to repair one or both openings from the cavity. Second, even if we repaired only the defect of the right side, the incision close tension was lower than the left ventricular incision according to the Laplace law, because the radius of the new cavity was smaller than left ventricular radius. Finally, the incision and suture affected both ventricles less. We repaired two cases with posterior VSR and ventricular septal dissection and one case with anterior VSR and ventricular septal dissection. The posterior location of the VSR has been reported to be associated with poor surgical outcomes because of right ventricular dysfunction, complex ruptures, and difficult repair (23). However, we observed no impact of VSR location on patient outcomes. In our study, posterior VSR was repaired in 3, 2, and 2 cases by right atria incision, ventricular septal dissection incision, and left ventricular incision, respectively. Only one of seven patients (14.3%) with posterior VSR repaired by left ventricular incision died of heart failure 2 months after surgery. The approach of right atrial incision or ventricular septal dissection incision was easier and less injurious than that of left ventricle incision and might be a cause of better results. More cases are needed to support our explanations.

There is debate on the impact of concomitant CABG on the prognosis of patients undergoing surgical repair for post-AMI VSR. Perrotta et al. (24) reviewed the scientific literature from 1950 to April 2009, and 18 papers were deemed relevant to the topic. Seven studies showed statistical evidence of benefit of concomitant CABG, especially with complete revascularization. Another five studies recommended CABG with VSR, even in the absence of statistical evidence. These studies showed a mortality decrease from 26.3% without revascularization down to 21.2% with revascularization and an actuarial survival at 5 years from 29% up to 72%. Based on this, it was concluded that for patients undergoing concomitant CABG to all the stenotic coronary arteries, supplying the non-infarcted area was associated with both improved 30-day survival and long-term survival. However, six out of 18 studies did not find any difference. Regarding operative outcomes, the data from the Society of Thoracic Surgeons showed that concomitant CABG did not reduce the risk of operative death (4). Based on our study's findings, the mortality increased with the number of diseased coronary vessels, although there was no statistical significance. This, however, suggested that the extent of coronary artery disease might be associated with outcome. Thus, we could conclude that concomitant CABG was safe because the number of grafts and the use of LIMA was not associated with all-cause death, but the aortic cross time did not increase significantly. In patients with VSR, a generalized lack of collateral flow between the coronary arteries effectively separates regions of the myocardium, and the additional blood provided by bypassing may help to overcome the lack of collaterals (25). Therefore, we preferred to perform bypassing for all diseased vessels including the culprit vessel, which is occasionally entrapped in the suture line of the ventriculotomy.

The limitations of this study are its retrospective design and limited sample size. Larger sample size and more detailed analyses are required to support our results.

Treatment strategies for VSR should be based on the patient's condition and comprehensively determined using real-time evaluation and monitoring. Surgical treatment resulted in good outcomes for patients with VSR after AMI. Delaying VSR repair beyond 14 days after rupture was optimal for decreased mortality rates and residual shunts. RBC transfusion and the NT-proBNP level were independent risk factors for mortality.

## Data availability statement

The raw data supporting the conclusions of this article will be made available by the authors, without undue reservation.

## Ethics statement

The studies involving human participants were reviewed and approved by Ethics Committee of the General Hospital of Northern Theater Command. Written informed consent for participation was not required for this study in accordance with the national legislation and the institutional requirements.

## Author contributions

KZ, DT, HJ, and HW contributed to the study design and analysis, conducted the study, and performed the examinations. BL and BS contributed to the collection and assembly of data. KZ, BL, and HW performed the statistical analysis, wrote the manuscript, and provided final approval of the manuscript. All authors have read and approved the final version of the manuscript.

## Funding

This study was supported in part by Liaoning Province, Science and Technology public relations project of China (Grant No. 2020JH2/10300170).

## Conflict of interest

The authors declare that the research was conducted in the absence of any commercial or financial relationships that could be construed as a potential conflict of interest.

## Publisher's note

All claims expressed in this article are solely those of the authors and do not necessarily represent those of their affiliated organizations, or those of the publisher, the editors and the reviewers. Any product that may be evaluated in this article, or claim that may be made by its manufacturer, is not guaranteed or endorsed by the publisher.
